# Estradiol-17β Pharmacokinetics and Histological Assessment of the Ovaries and Uterine Horns following Intramuscular Administration of Estradiol Cypionate in Feral Cats

**DOI:** 10.3390/ani10091708

**Published:** 2020-09-21

**Authors:** Timothy H. Hyndman, Kelly L. Algar, Andrew P. Woodward, Flaminia Coiacetto, Jordan O. Hampton, Donald Nickels, Neil Hamilton, Anne Barnes, David Algar

**Affiliations:** 1 School of Veterinary Medicine, Murdoch University, Murdoch 6150, Australia; kelly_algar@hotmail.com (K.L.A.); F.Coiacetto@murdoch.edu.au (F.C.); jordan.hampton@murdoch.edu.au (J.O.H.); a.barnes@murdoch.edu.au (A.B.); 2 Faculty of Veterinary and Agricultural Sciences, University of Melbourne, Melbourne 3030, Australia; andrew.woodward@unimelb.edu.au; 3 Lancelin Veterinary Hospital, Lancelin 6044, Australia; nickelsd@bigpond.net.au; 4 Department of Biodiversity, Conservation and Attractions, Locked Bag 104, Bentley Delivery Centre 6983, Australia; neil.hamilton@dbca.wa.gov.au (N.H.); dave.algar@dbca.wa.gov.au (D.A.)

**Keywords:** conservation, invasive species, mathematical modelling, pharmacology, reproduction, wildlife

## Abstract

**Simple Summary:**

Feral cats (*Felis catus*) have a devastating impact on Australian native fauna. Several programs exist to control their numbers through lethal removal, using tools such as baiting with toxins. Adult male cats are especially difficult to control. We hypothesized that one way to capture these male cats is to lure them using female cats. As female cats are seasonal breeders, a method is needed to artificially induce reproductive (estrous) behavior so that they could be used for this purpose year-round (i.e., regardless of season). Estrogens can be given to cats to induce estrous behavior, but it is not known for how long this elevates the blood concentrations of estradiol. Following the administration of a long-acting estrogen, estradiol cypionate, to nine captive feral cats, blood estrogen concentrations remained elevated for several days. This information will be useful to ongoing studies that are investigating ways to reduce the abundance of feral cats in Australia, especially adult male cats.

**Abstract:**

The control of feral cats (*Felis catus*) in Australia is a key biological conservation issue. Male cats are more difficult to control than female cats. Collared and tagged female cats displaying estrous behavior have been considered as a way to lure male cats and reveal their locations. As female cats are seasonal breeders, artificial induction of estrous behavior following the administration of a long-acting estrogen could improve their use for this purpose. Estradiol cypionate was intramuscularly administered to nine entire non-pregnant female feral cats, of unknown estrous status, at 0.1, 0.3, or 0.5 mg/kg. Mean peak serum concentrations of estradiol-17β were 365 pg/mL (0.1 mg/kg), 1281 pg/mL (0.3 mg/kg), and 1447 pg/mL (0.5 mg/kg). The time-course of estradiol-17β concentrations after various doses of estradiol cypionate was assessed using non-compartmental and non-linear mixed-effects methods. At the highest-studied dose (0.5 mg/kg), the 50th percentile of estradiol-17β concentrations exceeded 0.1 ng/mL for 11.8 days, and 0.05 ng/mL for 14.6 days. The duration increased with increasing dose. No signs of toxicity were noticed in any cat during the study. This information will be useful to ongoing studies that are investigating ways to reduce the abundance of feral cats in Australia, especially adult male cats.

## 1. Introduction

Control of feral cats (*Felis catus*) is considered a key conservation issue in Australia due to the threat they pose to many endangered animal species through predation and disease transmission [1]. Many Australian conservation organizations use broadcast sodium fluoroacetate (compound 1080) poison baits to kill feral cats [2,3]. It is rare (or undocumented) for all feral cats to be killed by a baiting program. Surviving individuals invariably consist predominantly of males that are larger and more adept at hunting and less likely to consume baits [3]. Targeting male cats for lethal control has therefore become a recent focus for feral cat control programs [4]. 

A cogitated approach for effective control of male cats that warrants consideration is to use reproductively cycling female cats as a lure. A number of methods to attract feral cats have been assessed and scent-based lures (anal gland preparations) were found to be the most effective [5]. 

A cycling female cat could attract a male cat and reveal its location by acting as a “Femme Fatale”. A Femme Fatale is analogous to a ‘Judas’ animal that is utilized to locate and kill individual animals of gregarious invasive species [6,7,8]. The Judas animal control technique was originally developed for feral goats (*Capra hircus*) [9], and this method has been enhanced through the use of Mata Hari female goats where, following tubal ligation, female goats are administered exogenous estrogen so they more actively seek out, and are searched for by, male goats [10,11]. 

The reproductive cycle of the cat is seasonal, and where a captured feral cat is in her estrous cycle is difficult to determine without considerable handling for assessment. Estrous behavior in the cat can be induced following the administration of exogenous estradiol-17β [12,13,14,15,16,17]. In a variety of mammalian species, including cats, there is a latency period of approximately 1–3 days between drug administration and the onset of estrous behavior that does not appear to be dependent on the dose administered [12,14,18,19,20]. Higher doses of estradiol-17β have been associated with longer periods of estrous behavior [19,21,22] and sustaining elevated blood concentrations of estradiol-17β can be achieved through the regular administration of estradiol-17β [12] or by giving custom-made subcutaneous implants of estradiol-17β [13,23]. An alternative and simple approach, more appropriate for use in a feral animal where minimal handling is required, could be to administer a single dose of a longer-acting preparation of estradiol-17β that is commercially available. 

Numerous semisynthetic estrogens can confer a long duration of action. Commonly used preparations include estradiol cypionate, estradiol benzoate, and estradiol valerate [24,25], with estradiol cypionate having the longest duration of action [26,27]. Following the intramuscular injection of estradiol-17β esters (e.g., cypionate) in oily solutions, the oils are absorbed leaving a microcrystalline depot of estradiol cypionate at the injection site that is released into the circulation at a slow rate [23,28]. The disposition of radiolabeled (^14^C) estradiol-17β has been investigated in cats [29] but we are unaware of any studies on the disposition of estradiol-17β following the administration of estradiol cypionate in this species.

Estradiol cypionate has been used to stimulate estrous behavior in female cats [14,30] but due to concerns about estrogen toxicity, modern recommendations for the use of this drug in domestic cats are limited to pregnancy termination [24,25,31,32]. While assessing the persistence of behavior in feral animals is challenging, knowledge of the pharmacokinetics of estradiol cypionate in feral cats will assist the Femme Fatale technique by providing information on the length of time that supraphysiological concentrations of estradiol-17β persist for in cats post-injection. This information can be used in concert with the histological assessment of the ovaries and uteri to correlate the association between blood estradiol-17β concentration and the activity of the reproductive tract. The aim of this study was therefore to assess the disposition of estradiol-17β and the histological appearance of the ovaries and uteri in feral cats following the intramuscular administration of estradiol cypionate. This information could then be used in future behavioral and field studies. Subsequent field studies could then assess the efficacy of Femme Fatales in attracting male cats (e.g., through the use of cameras mounted to female cats) [33].

## 2. Material and Methods

### 2.1. Ethics Statement

Approval of our experimental work was provided by the animal ethics committees of Murdoch University (R3087/18) and the Department of Biodiversity, Conservation and Attractions (DBCA) (2018-25F). Both of these committees adhere to the Australian Code for the Care and Use of Animals for Scientific Purposes (2013). The experiment was carried out in January of 2019.

### 2.2. Study Animals

Nine mature, female feral cats (mean weight ± SE = 3.1 kg ± 0.32 kg; range = 2.4–3.6 kg) were captured at rural effuse sites (rubbish tips) in Western Australia, using Sheffield wire small cage traps (Sheffield Wire Products, Australia) [34]. Cats were transported to the Wildlife Research Centre of DBCA in Perth in their capture cages on the morning of capture.

### 2.3. Housing

Before the trial commenced, the cats were habituated to captivity for a period of one month. During this time, the cats were housed in groups of three in outdoor pens (3.0 × 5.0 × 2.0 m) constructed of cyclone wire. Each pen contained a medium-sized wooden kennel (80 × 60 × 60 cm; Allpet Products, Welshpool, Australia) to provide shelter. Enrichment included branches for scratching and climbing, and PVC pipes for hiding. Tinned cat food and dried cat biscuits were supplied daily to each animal and water was available ad libitum. Human interaction was restricted to pen cleaning and daily monitoring of the animals’ health. Monitoring included assessments of each animal’s mentation, appearance, gait, respiratory rate and effort, water and food intake, and fecal and urine output.

### 2.4. Trial

Each cat was randomly assigned to one of three groups to receive 0.1, 0.3, or 0.5 mg/kg of intramuscular estradiol cypionate (Depo-Estradiol^®^, Pfizer, NY, USA, 5 mg/mL).

The cats were relocated to smaller trial cages measuring 1.0 × 3.0 × 2.3 m. These cages had concrete floors and a litter tray, but otherwise, they were furnished the same as the pre-trial housing. On the first day of the study (day 0), each cat was physically captured and then sedated using a mixture of medetomidine (60 µg/kg, Domitor^TM^, Zoetis Australia, West Ryde, Australia, 1 mg/mL) and butorphanol (0.2 mg/kg, Torbugesic^®^, Bayer, Pymble, Australia, 10 mg/mL) that was injected intramuscularly. This was followed 1–5 min later by an intramuscular injection of ketamine (5 mg/kg, Ketalar^TM^, Zoetis Australia, 100 mg/mL). Once sedated, each cat was weighed, and blood was collected from its jugular vein. Each cat was then injected with the assigned dose of estradiol cypionate. The animals were then monitored for 1–3 h before being returned to their trial cages.

The cats were re-captured, with nets or traps, from their trial cages for blood sampling. Blood was collected from alternating jugular veins on days 1, 2, 4, 6, 8, and 10. At each occasion, the cat was sedated with medetomidine, butorphanol, and ketamine and approximately 2 mL of blood was collected as described above. After blood sampling, all cats were given atipamezole (200 µg/kg, Antisedan^TM^, Zoetis Australia, 5 mg/mL) via intramuscular injection. The cats were closely observed for 1–3 h after the sampling before being returned to their trial cages.

Whole blood samples were centrifuged at 2500 *g* for 10 min immediately following sampling. A minimum of 0.5 mL of serum was submitted overnight to a pathology laboratory (Gribbles-Victoria, Australia) in foam cooler boxes containing ice. The serum samples were processed within 24 h.

The concentration of estradiol-17β in each serum sample was determined by ELISA using the ADVIA Centaur Estradiol-6 III assay. The linear range of this assay is reported by the manufacturer (Siemens, New York, NY, USA) as 0.007–1.0 ng/mL (25.7–3,670 pM), and using human serum samples with approximately 200–3400 pmol/L of estradiol-17β, the coefficient of variation has been externally assessed as being 8–24% [35]. For each serum sample with a concentration of estradiol-17β greater than 1 ng/mL (1000 pg/mL), the serum was diluted so that the concentration was within the linear range of the assay. The dilution factor was then used to determine the concentration of estradiol-17β in these samples.

At the completion of the trial, on day 10 after estradiol administration, cats were sedated using the methods described above and then euthanased by intravascular or intracardiac injection of 160 mg/kg of pentobarbitone (Lethabarb^TM^ Euthanasia Injection, Virbac, Sydney, Australia, 325 mg/mL).

### 2.5. Histology

Following euthanasia, samples of ovary and uterine horn from each cat were collected and fixed in 10% (v/v) neutral buffered formalin for histological examination. The fixed sections were blocked, embedded in paraffin, cut at 4 μm, and stained with haematoxylin and eosin. The slides were examined by a specialist veterinary pathologist who was blinded to the origin of the sections.

The reproductive tracts were histologically characterized into the stage of cycle based on the presence or absence of the ovarian follicles, corpora lutea, corpora albicans, and the appearance of the endometrium. Sections of ovary were examined for the presence or absence of primary, secondary, early tertiary and Graafian follicles, corpora lutea, and corpora albicans [36]. Corpora lutea were categorized according to an established luteal pseudopregnancy staging system [37] as either pseudopregnancy stage 1 (PP1), PP2, PP3, PP4.1, or PP4.2. Briefly, corpora lutea with elongated-to-round-shaped luteal cells with moderate numbers of small lipid droplets finely organized on the cell periphery (classified as type I vacuolation) were categorized as PP1. Corpora lutea with polyhedral-shaped luteal cells and moderate-to-heavy type I vacuolation were categorized as PP2. Corpora lutea with polyhedral-shaped luteal cells and moderate-to-heavy vacuolation, with large vacuoles that were coarsely scattered throughout the cell (classified as type II vacuolation), were categorized as PP3. Corpora lutea with irregularly-shaped luteal cells and heavy type II vacuolation were categorized as PP4.1. Corpora lutea with signet ring-shaped luteal cells with heavy type II vacuolation were categorized as PP4.2.

Sections of uterine horn were examined and classified using an established system [38]. The endometrium was classified as either a single, pseudostratified, or hyperplastic epithelial lining. The endometrial glands were classified as having either a single cell lining, pseudostratified lining, or hyperplastic lining with or without glandular secretion and dilatation, cystic changes, and/or proliferation of the endometrial glands.

### 2.6. Non-Compartmental Pharmacokinetic Calculations

The maximum serum concentration (*C_max_*) of estradiol-17β following intramuscular administration was the highest measured concentration for each animal. The time at *C_max_* (*t*_max_) was also recorded. The terminal rate constant (*λ*_z_) was calculated as the negative slope of the semi-logarithm plot of each animal created from the terminal time points (t = 2, 4, 6, 8, and 10 days). Either the last four or five time points were used depending on which negative slope was supported by the higher R^2^ value. The terminal half-life (*t_1/2β_*) was calculated as ln(2)/*λ*_z_. The area under the serum concentration time curve (*AUC_0→__∞_*) was estimated by the trapezoidal rule extrapolated to infinite time. The non-compartmental calculation of the volume of distribution [39] at pseudo-equilibrium was not calculated because it relies on the rate constant, *λ*_z_, being an elimination rate constant. Given that the drug disposition following intramuscular administration follows flip-flop (absorption-limiting) kinetics, this assumption is invalid.

### 2.7. Pharmacokinetic Modelling

A non-linear mixed-effects (NLME) model [40] was defined in Monolix (Lixoft, Antony, France). The structural model was a one-compartment mammillary model of the form:

(1) $$\frac{dA}{dt}=\;-k_{a}.A$$

(2) $$\frac{dX}{dt}=k_{a}.A-k_{e}.X$$

(3) $$k_{e}=\left(C_{1}+k_{a}\right)$$

(4) $$X_{\left(t=0\;\right)}=\left(B_{X}.V_{C}\right)$$

where absorption and elimination were both first order, and the starting estradiol-17β concentration was implemented as an initial condition for the central compartment. The one-compartment structure was selected after observation of scattered log-transformed concentration-time data. The parameters to be estimated were:

*k_a_*: Absorption rate constant
*V_C_*/F: Central compartment volume of distribution
*C*_1_: Difference between absorption and elimination (*k_e_*) rate constants
*B_X_*: Baseline estradiol-17β concentration
σ_ε_: Residual error standard deviation

The statistical model specified log-normal distributions of the parameters, and a proportional error model. As all parameters are therefore positively valued, in this model, the absorption rate constant must be smaller than the elimination rate constant (Equation (3); [41]). A random effect (between-subject variability) was not estimated for the volume of distribution, as initial analysis suggested that the data were not sufficient to support this.

Goodness-of-fit of the model was assessed by visualization of the observed and predicted estradiol-17β concentrations, the relative standard errors (RSE%) of the parameter estimates, and the Bayesian information criterion (BIC).

Two estimates of a supraphysiological serum concentration of estradiol-17β were chosen for simulations: 0.1 ng/mL and 0.05 ng/mL. These estimates were based on the natural cycles of estradiol-17β in domestic cats [42,43]. To determine the predicted time at which the proposed threshold concentrations of interest would be reached, simulations were conducted from the final pharmaco-statistical model. Simulations of 2000 subjects (for each dose) were performed using the ‘Simulx’ package in R (version R-3.6.3) [44]. A range of doses, including and above the studied doses, was examined, and the time for which 10%, 50%, and 90% of simulated subjects exceeded the target concentrations was determined. The prediction intervals were obtained across the simulated time series, and interpolating splines used to determine the time-to-reach-target.

## 3. Results

No evidence of ill-health was detected in any cat for the duration of this study.

**Figure 1:**
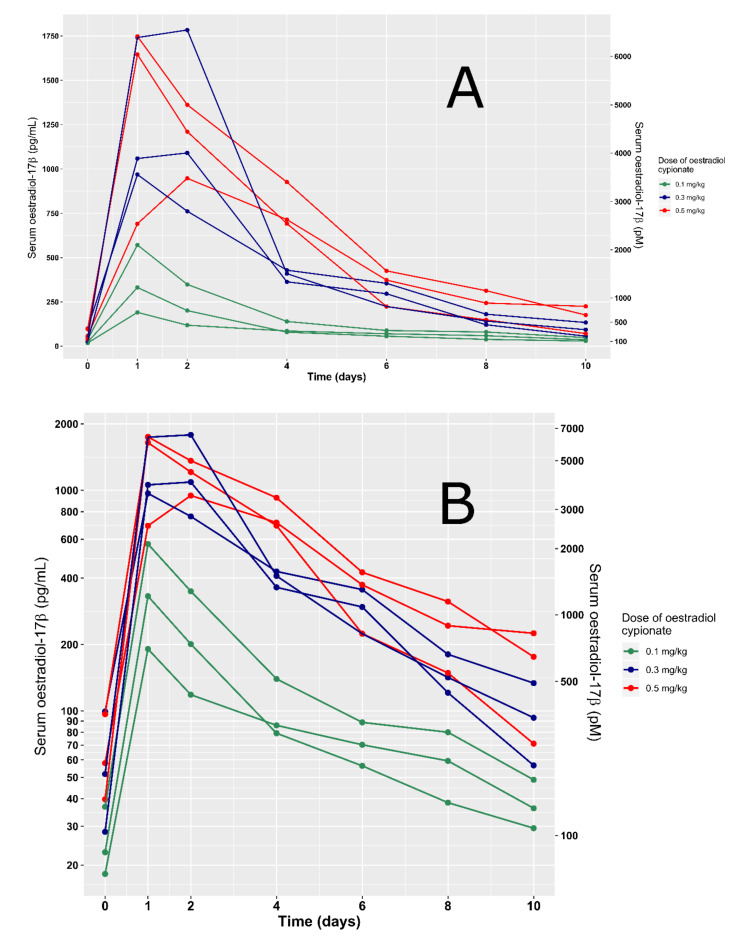
Observed serum concentration of estradiol-17β following the intramuscular administration of 0.1–0.52 mg/kg of estradiol cypionate to nine feral cats. Linear (**A**) and log10 scales (**B**).

Higher doses of estradiol cypionate were generally associated with higher maximum serum concentrations of estradiol-17β (Figure 1) and larger areas under their respective serum concentration time curves (Table 1).

**Figure 2:**
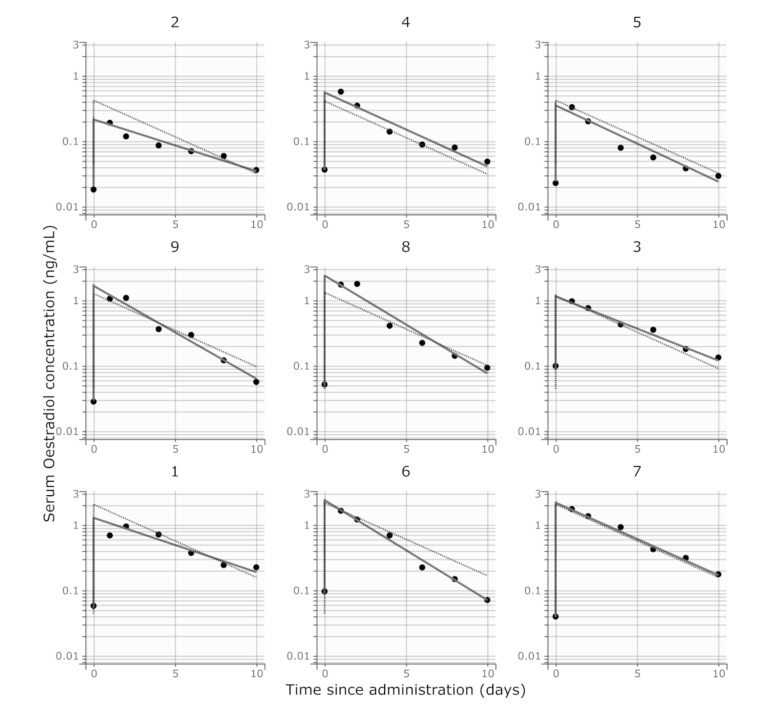
Individual goodness-of-fit plots for the estradiol-17β concentrations after intramuscular administration of estradiol cypionate. Subjects 2, 4, and 5: nominal dose 0.1 mg/kg (of estradiol cypionate); subjects 9, 8, and 3: nominal dose 0.3 mg/kg; subjects 1, 6, and 7: nominal dose 0.5 mg/kg. Points are the individual observations. Solid lines are the individual predictions. Dashed (light grey) lines are the population predictions (fixed effects only).

**Figure 3:**
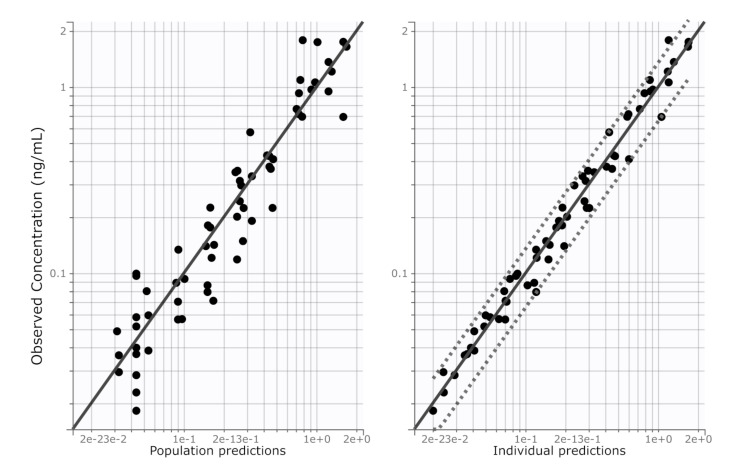
Identity (observations vs. predictions) plots for serum estradiol-17β concentrations on logarithmic axes. The left panel is the population predictions (fixed effects), and the right panel is the individual predictions (fixed and random effects). The solid line is the line of identity (y = x). This model uses proportional residual error.

The final model with proportional error variance, log-normal distributions of the pharmacokinetic parameters, and baseline estradiol-17β concentrations as the initial model condition was a reasonable fit to the available data (Figure 2). Each subject’s data was closely predicted (Figure 3) and there was no apparent model failure across the studied doses. The major apparent weakness of the final model was difficulty obtaining a smooth curve prior to the first observation at day 1, presumably due to the lack of information in the data regarding this phase. The parameter estimates obtained from the final model are described in Table 2. The size of the residual error was small (*b* = 0.21).

Parameters were precisely estimated with relative standard error (RSE%) of the fixed effects ranging from 2.5 to 18%. Though RSE% were small, the estimates for the elimination rate and volume of distribution were highly correlated (*R:* −0.567), suggesting that these parameters were not individually identifiable. This is unsurprising considering that the terminal phase, representing most of the available data, is dominated by the absorption process in this case.

**Figure 4:**
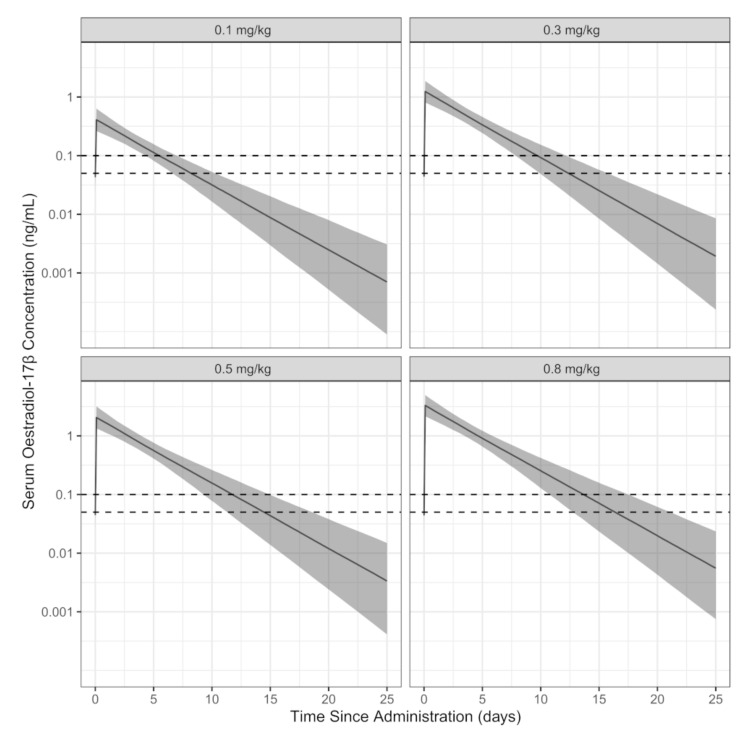
Predicted time-course of estradiol-17β concentrations after simulated administration of 0.1, 0.3, 0.5, or 0.8 mg/kg intramuscular estradiol cypionate from the final pharmaco-statistical model. Each dose comprises 2000 simulated subjects. The dark solid line is the median concentration. The band represents, from top-down, the 90th and 10th percentile concentrations. The dashed lines represent 0.1 (upper) and 0.05 (lower) ng/mL of estradiol-17β.

From the final model, a simulated population was used to determine the predicted duration that threshold concentrations (100 pg/mL and 50 pg/mL) were exceeded, after various doses (Figure 4). The durations for the 10th, 50th (median), and 90th percentiles are shown in Table 3. As expected, the time-to-reach target increased with increasing dose. Most of these estimated times, particularly for target 0.05 ng/mL, were extrapolated beyond the observation times from the experiment.

**Figure 5:**
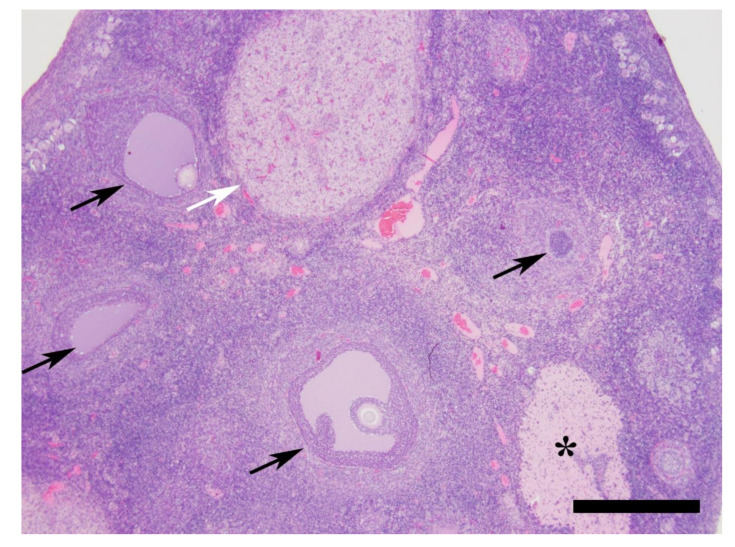
Feline ovary. The ovary contains a corpus luteum (white arrow), a corpus albicans (asterisk), and ovarian follicles at various developmental stages (black arrows). Hematoxylin & Eosin (H&E) stain. Bar = 500 µm.

**Figure 6:**
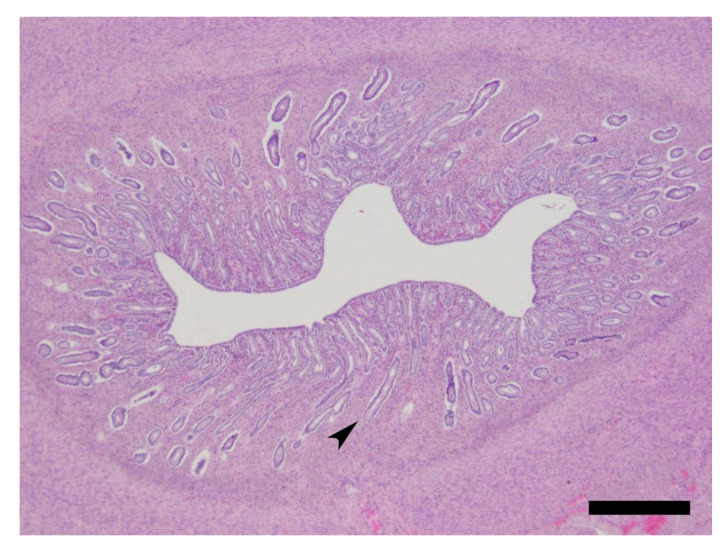
Feline uterine horn. The endometrium demonstrates elongated long glands (arrowhead), which are characteristic of the luteal stage. H&E stain. Bar = 500 µm.

The reproductive tracts from all cats were histologically consistent with luteal phase, ranging from PP1 to PP3/4 (Figure 5 and Figure 6, Table 4). The sections of ovaries from all cats contained one to multiple corpora lutea in various phases of development and regression, numerous follicles in varying phases of development raging from primary to tertiary follicles, and an ovary from a single cat contained a single corpus albicans. The uteri from eight of the nine cats were also histologically consistent with the luteal phase with hyperplastic endometrium and numerous elongated endometrial glands.

## 4. Discussion

This study has provided the first report of the disposition of estradiol-17β and the histological appearance of the ovaries and uteri in female feral cats of unknown cycle stage, following the intramuscular administration of estradiol cypionate.

In our study, the pre-drug serum concentrations of estradiol-17β ranged from 18.2 to 96.7 pg/mL (mean = 50.2 pg/mL, 95% CI = 27.2–73.2 pg/mL). These concentrations are similar to those reported in investigations of domestic cats. In one study, the mean peak and trough plasma concentrations of endogenous estradiol-17β during polyestrous in four naturally cycling female cats were 59.5 pg/mL and 8.1 pg/mL, respectively [43]. In a separate study, the peak plasma concentrations of estradiol-17β in domestic cats were 50–70 pg/mL [42].

Other studies have provided insights into the association between blood concentrations of estradiol-17β and estrous behavior and/or mating. In a previous study [42], the follicular phase was defined by plasma estradiol-17β concentrations that exceeded 20 pg/mL and using that definition, 100% of cats (*n* = 23) displayed estrous behavior by the sixth day of the follicular phase, but the percentage steadily declined over the next six days. That is, sexual behavior was still seen in some cats outside of the follicular phase; plasma concentrations of estradiol-17β were approximately 8–10 pg/mL at that time. In another study, male cats were observed to mate with female cats who had plasma estradiol-17β concentrations of 3.8–146 pg/mL (mean ± SE = 40.3 ± 15.6) [16]. In that same study, mating was also seen in female cats following the subcutaneous administration of 4 cm or 8 cm implants of estradiol-17β (the specific dose was not reported) that resulted in plasma estradiol-17β concentrations of 29 ± 2 and 48 ± 4 pg/mL, respectively.

Following the administration of 0.5 mg/kg of estradiol cypionate, we found that the mean number of days that serum concentrations of estradiol-17β exceeded 20 pg/mL was 17.8 days. Clearly, lower doses exceeded higher concentrations for shorter periods of time. For example, following 0.1 mg/kg of estradiol cypionate, the mean number of days above 100 pg/mL was only 3.9 days. A dose-independent latency of approximately 1–3 days between drug administration and the onset of estrous behavior has been described by others [12,14,18,19,20]. Even accommodating for this period of latency, it is reasonable to assume that estrous behavior in female feral cats would last for at least several days following the administration of higher doses (e.g., 0.5 mg/kg) of estradiol cypionate. This is consistent with other studies: another study [22] found that two days after the subcutaneous administration of 0.45 mg (total dose) of estradiol benzoate to female cats, the median duration of mating was 6.2 days. It was found that mating with male cats continued for less than a week after the cessation of daily subcutaneous administration of stilbestrol dipropionate or crystalline estradiol-17β to ovariectomized female cats [12]. Additionally, in a separate study, two 0.25 mg doses of intramuscular estradiol cypionate were administered 48 h apart to nine sexually demonstrative mixed-breed adult female cats [14]. Estrous behavior usually lasted for 14–28 days.

The persistence of supraphysiological plasma concentrations of estradiol-17β following the administration of estradiol cypionate has also been described in llamas (*Lama glama*) and humans, albeit at lower doses than what was used in our study. In a study on six sexually mature female llamas, supraphysiological plasma concentrations of estradiol-17β persisted for nine days following the intramuscular administration of 2.5 mg of estradiol cypionate [27]. In ten 20–35-year-old women, plasma concentrations of estradiol-17β did not return to baseline levels for 11.8 days after the intramuscular administration of 5 mg of estradiol cypionate [26]. In our study, and those by others [26,27], results were complicated by the presence of endogenous estradiol-17β. This challenge has been addressed in different ways.

One study [26] did not correct for the pre-dose plasma concentrations of estrogen, as the women in that study had been treated daily with oral contraceptives of 150 µg of levonorgestrel and 30 µg of ethinylestradiol. Treatment lasted for at least three months before the study commenced and continued for the duration of the study. This approach was intended to result in a relatively constant and low plasma concentration of estradiol-17β. Further, the anti-sera that was used to quantify plasma estradiol-17β cross-reacted by less than 0.1% with ethinylestradiol. Our study cats had not been treated with estrogens before our experiment commenced and could have been at any stage of their cycle, and so not surprisingly had a wide range of pre-dose serum concentrations of estradiol-17β. We decided to not subtract the baseline (endogenous) serum estrogen concentrations from the post-dose sample measurements for two reasons. First, the majority of serum estradiol-17β concentrations exceeded the baseline measurements substantially. In a study on oral estradiol cypionate in rats, this logic was used to leave post-dose serum concentrations of estradiol-17β unadjusted [47]. Although not stated explicitly, it appears that another study [27] did not adjust for physiological plasma concentrations of estradiol-17β in their pharmacokinetic calculations. The second reason why we chose not to adjust for baseline serum concentrations of estradiol-17β was that one of our later serum concentrations of estradiol-17β (from Cat 6) was below the pre-dose serum concentrations. Adjusting values in accordance with pre-dose serum concentrations could therefore result in negative serum estradiol-17β concentrations. A post-dose concentration that is lower than the pre-dose concentration could be due to negative feedback of exogenous estrogen on the gonadotropins in the pituitary gland; an effect that has been described in women [48], female sheep, [49] and female cats [50].

The ability to directly include the endogenous pre-study estradiol-17β concentration in a simple way is an advantage of the model-based approach. The reasonable fit of this model across concentrations, without a mechanism for ongoing endogenous estrogen release, supports the view that endogenous estrogen release was negligible during the observation window and that the observed estradiol-17β pharmacokinetics were dominated by the absorption rate. More advanced techniques for the treatment of endogenous analytes, such as those based on linear systems theory or deconvolution, could directly estimate the input of endogenous estradiol-17β as a function of time, but were considered beyond the scope of the current study and data.

A pharmaco-statistical (i.e., NLME) model is an ideal tool for statistical inference from pharmacokinetic data, as the model structure systematically accounts for variation between subjects, and information is borrowed across all doses and subjects simultaneously. The NLME model in this study met the objective of describing the time course of estradiol-17β concentrations as a function of dose. However, some limitations of the model are worth highlighting. The simulation of a large hypothetical population of cats is not intended to overcome the small sample size; it is instead a tool to explore, in detail, the information contained within the data, under specific assumptions. To apply the predictions to a true population rigorously, an external validation procedure would be needed. The primary limitation to the internal validity of those simulations from the current study is the reasonableness of the log-normal distribution selected for the absorption rate constant; this is difficult to evaluate with a small sample size. A key observation in the analysis of these data was that the volume of distribution and the elimination rate constant were difficult to estimate, presumably due to identifiability constraints. As the pharmacokinetic observations were dominated by the absorption rate (flip-flop phenomenon), the data are not very informative regarding these parameters. This could readily be resolved by the addition of intravenous data, and the NLME approach to analysis facilitates this. The estimates of these parameters from the current study are essentially arbitrary, and they should not be considered externally valid or used for any other purpose.

A liquid chromatography-mass spectrometry assay was recently developed for the specific detection of estradiol cypionate (as distinct from estradiol-17β) [46]. This was to address the complication of measuring endogenous and exogenous estradiol-17β concurrently, but obviously this has utility only to those studies using the cypionate ester of estradiol-17β. This assay is therefore more appropriate for drug monitoring than for use in studies intending to assess the biological effects of estradiol-17β.

In our study, some cats had peak serum concentrations of estradiol-17β that were over 1,600 pg/mL, which is more than an order of magnitude higher than concentrations measured in naturally cycling female cats [42,43]. Further, the highest dose we administered (0.5 mg/kg) is greater than the clinical doses of estradiol cypionate that are advocated for pregnancy termination in cats, which range from 0.25 mg/cat (~0.06 mg/kg for an adult cat; [24,31]) to 0.25 mg/kg [25]. Cats are reported to be more susceptible to the adverse side effects of estrogens than ferrets, rats, mice, and dogs [32]. Side effects of estradiol-17β include cystic endometrial hyperplasia, pyometra, and dose-dependent bone marrow toxicity leading to leukopenia, thrombocytopenia, and fatal aplastic anemia [24]. In some cases, the bone marrow depression may be transient with resolution beginning in 30–40 days [25]. Estradiol-17β has been associated with a minor decrease in aggression in ovariectomized and entire female cats as defined by a slightly (but statistically significant) prolonged latency time to attack anaesthetized rats [51]. In rats, estradiol cypionate was associated with a significant increase in vaginal counts of culturable bacteria three days after treatment [52]. Our histological assessment of the ovaries and uterus horns did not reveal any lesions, but our study did not otherwise investigate estradiol-17β toxicity. Nevertheless, no overt signs of illness were detected in any cat during daily monitoring for the 10 days following administration of estradiol cypionate.

In cats, daily doses of diethylstilbestrol between 0.1 mg/kg and 2–4 mg/kg are associated with average survival rates of 25–75 days [32]. At daily doses of 4–10 mg/kg, the average survival time reduces to 10–25 days [32]. Lethal single doses are typically defined at about 10–70 mg/kg [32]. It should be noted that estradiol-17β has been shown to be more potent than diethylstilbestrol in rats [53] and women [23] for a number of estrogenic-mediated responses, but their relative potency in cats is not described. It appears, based on the lack of any relevant observations, that the single doses of estradiol cypionate administered in this study (0.1–0.5 mg/kg) are below the total dose associated with death in cats, or that toxicity manifests clinically more than 10 days after drug administration.

Following the assessment of the uterus horns, we found that eight out of nine cats had minimal-to-moderate hyperplasia of the endometrium, which is consistent with the findings by Harris and Michael [13], where an increase in endometrial surface area was observed in ovariectomized female cats administered estrogen implants. All cats were in the luteal phase but a limitation of the histological aspect of our study is that we did not have a no-drug control group nor did we perform a suite of assessments of the reproductive tract before administering the drug (e.g., ultrasound, vaginal cytology, etc.). This would have aided in determining when the cats might have ovulated. Another limitation is that progesterone was not measured in the serum samples and so interpreting the histological appearance of the ovaries becomes problematic. The presence of luteal tissue may indicate a drug-induced diestrus, with production of progesterone. Behavioral effects of excessive progesterone are inhibitory; feral cats receiving an oral progesterone-type compound in food showed fewer estrous cycles, a lack of sexual interest and loss of ‘‘social status’’ [54]. This highlights the potential interaction of hormonal treatment with the natural reproductive cycle of the cats and indicates the need for further investigation of the behavior and hormonal effects when cats are in seasonal anestrus. In particular, future behavioral studies should consider ovariectomized cats. Without an ovarian response to the exogenous estrogen, the potentially confounding effect of progesterone would be removed.

## 5. Conclusions

This study has provided the first report of the disposition of estradiol-17β and the histological appearance of the ovaries and uterus horns in female feral cats following the intramuscular administration of estradiol cypionate. It was shown that supraphysiological serum concentrations of estradiol-17β persisted for several days following the administration of this long-acting estrogen. This will assist future behavioral studies and field investigations into the suitability of Femme Fatales for the management of male cats in feral cat control programs. Further work is needed to ascertain the impact these concentrations have on the behavior of female cats and for how long these female cats can be used as lures for adult male cats. Subsequent work would then be required to translate attracting male cats into effectively killing male cats that are attracted to Femme Fatale animals.

## Figures and Tables

**Table 1:** Pharmacokinetic parameters of estradiol-17β in feral cats and other species.

Study Animal Number/Population	Dose of Estradiol Cypionate (mg/kg)	Analyte Quantified in Assay	*C_max_*(ng/mL)	*t_max_* (Day)	*λ*_z_ (/Day)	*t_1/2β_* (Day)	*AUC_0→__∞_* (ng.Day/mL)	Reference
**2**	0.10	Estradiol-17β	0.191	1	0.137	5.06	1.11	this study
**4**	0.10	Estradiol-17β	0.571	1	0.163	4.25	2.08	this study
**5**	0.10	Estradiol-17β	0.331	1	0.168	4.13	1.20	this study
**3**	0.28	Estradiol-17β	0.969	1	0.217	3.19	4.84	this study
**8**	0.31	Estradiol-17β	1.784	2	0.245	2.83	6.47	this study
**9**	0.30	Estradiol-17β	1.091	2	0.351	1.98	4.49	this study
**1**	0.47	Estradiol-17β	0.947	2	0.198	3.51	6.17	this study
**6**	0.52	Estradiol-17β	1.646	1	0.360	1.92	5.91	this study
**7**	0.47	Estradiol-17β	1.748	1	0.259	2.68	8.00	this study
**Sexually-mature female llamas (*n* = 6)**	2.5 mg/llama (~0.023 mg/kg) im	Estradiol-17β	0.043 ^a^ (±SD = 0.002)	1	n.p.	n.p.	0.32 ^b^ (±SD = 0.024)	[27]
**Female cows (*n* = 5)**	10 mg/cow (~0.017 mg/kg) ^c^ im	Estradiol-17β	0.087 ^a^ (range: 0.057, 0.128)	1.66 (range: 0.54, 4.96)	n.p.	n.p.	n.p.	[45]
**20–35 year old women (*n* = 10)**	5 mg/woman im	Estradiol-17β	0.338 ^a,d^ (95% CI: 0.208, 0.550)	3.9 ^d^ (95% CI: 2.4, 6.2)	n.p.	n.p.	n.p.	[26]
**22–41 year old women (*n* = 12)**	5 mg/woman ^e^ im	Estradiol-17β cypionate	0.14 (±SD = 0.08)	0.70 ^f^ (±SD = 0.88)	0.288 ^f^ (±SD = 0.168)	3.74 ^f^ (±SD = 3.17)	0.71 ^f^ (±SD = 0.40)	[46]

*C_max_* = maximum serum/plasma concentration; *t_max_* = time of *C_max_*; *λ*_z_ = terminal rate constant; *t_1/2β_* = terminal half-life; *AUC_0→__∞_* = area under the serum concentration time curve from time = 0 to ∞; *Cl* = total body clearance. 95% CI = 95% confidence interval. n.p. = not presented. ^a^ Converted from pmol/L to ng/mL (or pmol.day/L to ng.day/mL) using a conversion factor of 0.00027238. ^b^ Calculated to the last measurable concentration. ^c^ The weight of each cow was estimated as 600 kg. ^d^ Data were presented by Oriowo et al. [26] as geometric means. The corresponding arithmetic means will be higher. ^e^ Administered concurrently with 25 mg of medroxyprogesterone acetate. ^f^ The units of time were converted from hours to days.

**Table 2:** Parameter estimates from the nonlinear mixed-effects (NLME) model.

	Parameter	Maximum Likelihood Estimate	Relative Standard Error (%)
**Fixed Effects**	*k*_a_	0.257	10.7
	C_1_	2.57 × 10^4^	11.1
	V_C_/F	1.63	2.54
	base_conc	0.0439	18
**Random Effects**	*k*_a_	0.251	31.9
	C_1_	0.229	32.8
	base_conc	0.516	25.7
**Error**	*b*	0.211	11.5

The random effects for ka, C0, and base_conc are log-normally distributed. No random effect was estimated for V_C_. C_1_ = *k*_e_-*k*_a_; V_C_ = central-compartment volume of distribution; base_conc: the endogenous estradiol-17β concentration at study start; *b* = residual error standard deviation (proportional scale).

**Table 3:** Predicted population time-to-reach-concentration (days), for the target estradiol-17β serum concentrations of 0.1 ng/mL and 0.05 ng/mL, after administration of various doses of intramuscular estradiol cypionate. For each dose, 2000 subjects were simulated from the final pharmaco-statistical model, including residual error in the predicted concentrations. The times to reach the target concentrations were determined from interpolating splines fitted to the empirical percentiles.

Dose of Estradiol Cypionate	Predicted Population Time-to-Reach-Concentration (Days)		
	10th Percentile	Median	90th Percentile
***Target 0.1 ng/mL (100 pg/mL)***			
**0.1 mg/kg**	4.28	5.44	6.90
**0.3 mg/kg**	7.76	9.83	12.1
**0.5 mg/kg**	9.40	11.8	14.9
**0.8 mg/kg**	10.7	13.8	17.5
***Target 0.05 ng/mL (50 pg/mL)***			
**0.1 mg/kg**	6.52	8.22	10.3
**0.3 mg/kg**	9.86	12.6	15.8
**0.5 mg/kg**	11.4	14.6	18.5
**0.8 mg/kg**	12.7	16.6	21.3

**Table 4:** Histological characterization and staging of ovaries and uterine horns.

Cat ID	Ovary Primary Follicles	Ovary Secondary Follicles	Ovary Early Tertiary Follicles	Ovary Graafian Follicles	Ovary CL	Ovary CA	Endometrial Surface Epithelium ^a^	Endometrial Glands ^b^	Stage of Cycle ^c^
1	Numerous	Numerous	Multiple	None	Multiple PP3	None	S to PS HC with MOD HYE	NLG with B, MIN D and E	Luteal/PP3
2	Numerous	Numerous	Multiple	None	Multiple PP3	None	S to PS HC with MIL HYE	NLG with B, MIN D and E	Luteal/PP3
3	Numerous	Numerous	Multiple	None	Multiple PP1	None	PS HC with MOD HYE	NLG with B, MOD D and E	Luteal/PP1
4	Numerous	Numerous	Multiple	None	Multiple PP3/4	None	S to PS HC with MOD HYE	NLG with B, MIN D and E	Luteal/PP3/4
5	Numerous	Numerous	Multiple	None	Multiple PP3	None	S to PS HC with MOD HYE	NLG with B, MIL D and E	Luteal/PP3
6	Numerous	Numerous	Multiple	None	Multiple PP3	None	S to PS HC with MIL HYE	NLG with B, MIL D and E	Luteal/PP3
7	Numerous	Numerous	Multiple	None	Multiple PP2/3	One	S to PS HC with MIL HYE	NLG with B, MIN D and E	Luteal/PP2/3
8	Numerous	Numerous	Multiple	None	Single PP2	None	S to PS C	FLG with E	Luteal/PP2
9	Numerous	Numerous	Multiple	None	Multiple PP3	None	S to PS HC with mil HYE	NLG with B, mil D and E	Luteal/PP3

Abbreviations: CL = corpora luteum, CA = corpora albicans, PP = pseudopregnancy. ^a^ S = simple, PS = pseudostratified, H = high, C = columnar, MOD = moderate, MIL = mild, HYE = hyperplasia. ^b^ NLG with B = numerous long glands with branching, FLG = few long glands, MIN = minimal, MOD = moderate, D = dilation, E = extension to base of endometrium. ^c^ See Amelkina et al. [37] for definitions of PP1–PP4.
